# Right ventricular restriction in interventional lung assist for acute respiratory distress syndrome

**DOI:** 10.1186/cc12109

**Published:** 2013-03-19

**Authors:** G Tavazzi, M Bojan, S Canestrini, M White, S Price

**Affiliations:** 1 University of Pavia Foundation Policlinico San Matteo IRCCS, Pavia, Italy; 2 Royal Brompton Hospital, London, UK

## Introduction

Acute cor pulmonale (ACP) is associated with increased mortality in patients ventilated for acute respiratory distress syndrome (ARDS). Interventional lung assist (iLA) allows a lung-protective ventilatory strategy, whilst allowing CO_2 _removal, but requires adequate right ventricular (RV) function. RV restriction (including presystolic pulmonary A wave) [1] is not routinely assessed in ARDS.

## Methods

A prospective analysis of retrospectively collected data in patients with echo during iLA was performed. Data included epidemiologic and ventilatory factors, LV/RV function, evidence of RV restriction and pulmonary hemodynamics. Data are shown as mean ± SD/median (interquartile range).

## Results

**Figure 1:**
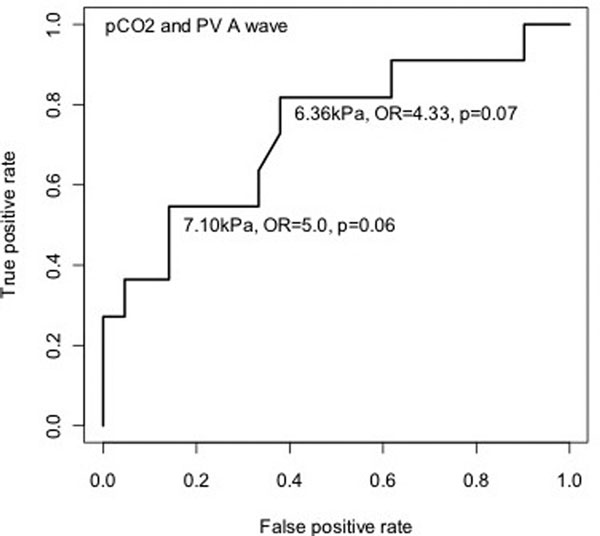


Thirty-two patients (45 ± 17 years), 22 male (68%), SOFA score 11.15 ± 2.38 were included. Pulmonary hypertension (PHT) was 53%, and hospital mortality 43%. Mortality was not associated with age, days on iLA, length of ICU stay, inotropic support, nitric oxide or level of ventilatory support, but was associated with pressor requirement (*P *= 0.005), a worse PaO_2_:FiO_2 _ratio (9.4 (7.8 to 12.6) vs. 15.2 (10.7 to 23.9), *P *= 0.009) and higher pulmonary artery pressures (56.5 mmHg (50 to 60) vs. 44.5 (40.5 to 51.2), *P *= 0.02). No echo features of ACP were found, with no significant difference between RV systolic function, pulmonary acceleration time and pulmonary velocity time integral between survivors and nonsurvivors. The incidence of RV restriction was high (43%), and independent of PHT, RV systolic function and level of respiratory support, but correlated with CO_2 _levels (restrictive 7.1 kPa (7.4 to 8.0) vs. 6.1 (5.8 to 6.8), *P *= 0.03). See Figure 1.

## Conclusion

Typical echo features of ACP were not seen in this study, possibly because of the protective ventilatory strategies allowed by use of iLA. The incidence of RV restriction may reflect more subtle abnormalities of RV function. Further studies are required to elucidate RV pathophysiology in critically ill adult patients with ARDS.
